# The Prognostic Value of Blood-Based Biomarkers in Patients With Testicular Diffuse Large B-Cell Lymphoma

**DOI:** 10.3389/fonc.2019.01392

**Published:** 2019-12-10

**Authors:** Jing Yang, Xinli Guo, Jianqi Hao, Yiting Dong, Tao Zhang, Xuelei Ma

**Affiliations:** ^1^State Key Laboratory of Biotherapy and Cancer Center, Collaborative Innovation Center for Biotherapy, West China Hospital, Sichuan University, Chengdu, China; ^2^West China School of Medicine, West China Hospital, Sichuan University, Chengdu, China

**Keywords:** testis, lymphoma, prognosis, neutrophil, lymphocyte, platelet

## Abstract

**Objectives:** Previous studies have reported the prognostic value of neutrophil/lymphocyte ratio (NLR), platelet/lymphocyte ratio (PLR), lymphocyte/monocyte ratio (LMR), and systemic immune-inflammation index (SII). However, the prognostic performance of these indices in patients with testicular lymphoma has not yet been studied. This study was to systematically evaluate the role of NLR, PLR, LMR, and SII in predicting survival for patients with testicular diffuse large B-cell lymphoma.

**Methods:** In this study, 28 patients with testicular diffuse large B-cell lymphoma were enrolled. We performed univariate and multivariate analyses to assess associations of indices incorporating blood cell counts with progression-free survival (PFS) and overall survival (OS).

**Results:** The results of univariate analysis revealed that International Prognostic Index (IPI) score (*p* = 0.010, *p* = 0.034, respectively), NLR (*p* = 0.003, *p* = 0.025, respectively), and LMR (*p* = 0.004, *p* = 0.010, respectively) were significantly associated with PFS and OS. Lactic dehydrogenase (LDH) (*p* = 0.017), absolute neutrophil counts (*p* = 0.018), absolute monocyte counts (*p* = 0.001), and SII (*p* = 0.005) were significantly associated with the risk of disease progression, while ECOG performance status (*p* = 0.016) was shown to be related to the risk of death. In the multivariate analysis, NLR (HR 9.069, *p* = 0.001) and absolute monocyte counts (HR 37.076, *p* = 0.001) were shown to be independently associated with risk for disease progression, while LMR (HR 0.077, *p* = 0.028), and ECOG performance status (HR 20.013, *p* = 0.026) were proved to be independent predictors of OS.

**Conclusions:** In conclusion, high absolute monocyte counts, high NLR and low LMR may indicate unfavorable prognosis in testicular diffuse large B-cell lymphoma patients. Since indices incorporating blood cell counts are low cost parameters, they may provide additional prognostic value beyond standard clinicopathological parameters. However, further studies are needed to confirm our findings.

## Introduction

Testicular lymphoma, a rare disease, accounts for 5–9% of testicular malignancies, 2% of extranodal lymphomas, and 1–2% of non-Hodgkin's lymphomas with an estimated incidence of 0.26 in 100,000 per year (1, 2). However, testicular lymphoma is the most common diagnosed testicular malignancy in men aged over 60 years (1, 3). By far, diffuse large B-cell lymphoma is the most common histological subtype, accounting for more than 80% of testicular lymphoma (1).

The survival of patients with testicular lymphoma has been gradually improving with the development of treatment strategies (4). A study including 769 patients with testicular diffuse large B-cell lymphoma has reported that the median overall survival is 4.6 years while the disease-specific survival rates at 3, 5, and 15 years are 71.5, 62.4, and 43.0%, respectively (5).

Several prognostic factors for testicular lymphoma patients have been reported, such as age, stage, tumor diameter, involvement of left testis, involvement of extranodal site, B symptoms, serum lactate dehydrogenase (LDH), serum β_2_-microglobulin, Eastern Cooperative Oncology Group (ECOG) performance status, and International Prognostic Index (IPI) score (2, 6). However, validated predictors for survival in patients with testicular lymphoma are still not available.

Previous studies have reported the prognostic value of blood cell counts in patients with several types of tumor (7, 8). In addition, the values of pertinent indices incorporating blood cell counts, i.e., neutrophil/lymphocyte ratio (NLR), platelet/lymphocyte ratio (PLR), lymphocyte/monocyte ratio (LMR), and systemic immune-inflammation index (SII) have also been reported to be associated with outcomes of those patients (9–11). However, to the best of our knowledgement, the prognostic significance of blood cell counts and indices derived from blood cells in patients with testicular lymphoma has not yet been studied.

In this study, we evaluated parameters at diagnosis including absolute lymphocyte counts, absolute neutrophli counts, absolute monocyte counts, absolute platelet count, NLR, PLR, LMR, and SII for their prognostic significance in patients with testicular diffuse large B-cell lymphoma.

## Materials and Methods

### Patients

We reviewed the clinical and laboratory data of lymphoma patients treated at West China Hospital between December 2008 and December 2016. Patients with pathological diagnosis of testicular lymphoma were included in this study and 45 patients were identified among the entire population. Patients were excluded if they had human immunodeficiency virus infection or severe coincident diseases, and then 34 patients with testicular lymphoma were identified. Finally, 28 testicular diffuse large B-cell lymphoma patients with follow-up data were enrolled in this research. This study was approved by the Ethics Administration Office of West China Hospital, Sichuan University before initiation.

We retrieved demographic data, medical records and laboratory results of those patients, including gender, age, B symptoms (fever, night sweating, or weight loss), Ann Arbor stage, site of testicular tumor, lymph node involvemen, extranodal involvement, LDH level, serum β2-microglobulin, Eastern Cooperative Oncology Group (ECOG) performance status, IPI, absolute neutrophl counts, absolute lymphocyte counts, absolute platelet counts, and absolute monocyte counts. All the patients were staged according to the Ann Arbor staging system. The five risk factors taken into account in the IPI are age, ECOG performance status, LDH level, Ann Arbor stage, and extranodal sites (12).

The methodology of this research is similar to our previously published manuscript (13). All the data of blood cell counts was collected within 10 days prior to diagnosis. NLR and PLR were calculated by dividing the absolute neutrophli counts and platelet counts by the absolute lymphocyte counts, respectively. LMR was defined as the absolute lymphocyte counts divided by the absolute monocyte counts. SII was calculated as platelet counts × neutrophil counts/lymphocyte counts.

### Statistical Analysis

Overall survival (OS) was defined as the period from the initial histological diagnosis to the time of last follow-up or until death from any cause. Progression-free survival (PFS) was defined as the interval between the date of initial histological diagnosis and the date of disease recurrences, progression, or death from any cause.

We divided patients into two groups according to the values of absolute neutrophil counts, absolute lymphocyte counts, absolute monocyte counts, absolute platelet counts, NLR, PLR, LMR, and SII, respectively, using the R package MaxStat (https://cran.r-project.org/web/packages/maxstat/index.html). The MaxStat package iteratively tests all possible cutpoints to find the cutpoint that achieves the maximum log-rank statistic (14, 15). The associations between each categorical variable and those indices were evaluated using the Fisher exact test and the Chi-square test, as appropriate. The associations between each continuous variable and those indices were evaluated using the Student's *t* test. As Kaplan–Meier method was used to estimate PFS and OS, the differences between groups was evaluated using the log-rank test. Univariate and multivariate analysis were carried out by the log-rank test and the Cox proportional hazard model. Variables with a *p* < 0.05 in the unvariate analysis were analyzed in the multivariate analysis to identify independent prognostic factors for patients with testicular diffuse large B-cell lymphoma. All tests were two-sided and a *p* < 0.05 was considered statistically significant. Statistical analyses were performed by R (version 3.6.1) and SPSS software (version 22.0, IBM Corporation, Armonk, NY, USA).

## Results

### Patient Cohorts and Characteristics

A total of 28 patients with testicular diffuse large B-cell lymphoma were identified and analyzed in this study. They had a median age of 65 years (range 37–84 years) at the time of diagnosis. Eleven patients (39.3%) were younger than 60 years while 17 patients (60.7%) were older than 60 years. Of the entire patients, 10 (35.7%) had tumor located on the left side, 17 (60.7%) on the right side, whereas only 1 (3.6%) on the bilateral sides. Half of the patients were diagnosed with Ann Arbor stage I-II testicular diffuse large B-cell lymphoma other half of patients were diagnosed at stage III-IV. B symptoms were identified in 4 (14.3%) patients. IPI score was calculated in all patients, with 0–1 in 13 (46.4%) patients and 2–5 in 15 (53.6%) patients. The baseline characteristics of the patients were shown in Table 1.

**Table 1:** Baseline characteristics of the study population (*n* = 28).

**Characteristics**	**Number of assessable patients (%)**
**Age (years)**	
Median (range)	65 (37–84)
≤ 60	11 (39.3%)
>60	17 (60.7%)
**Site of the primary tumor in the testicle**	
Left	10 (35.7%)
Right	17 (60.7%)
Bilateral	1 (3.6%)
**Ann arbor staging**	
I-II	15 (53.6%)
III-IV	13 (46.4%)
**B symptoms**	
Present	4 (14.3%)
Absent	24 (85.7%)
**Extranodal involvement (excluding testicular)**	
0	16 (60.7%)
1	8 (21.4%)
>1	4 (17.9%)
**IPI score**	
0–1	14 (50.0%)
2–5	14 (50.0%)
**ECOG PS**	
0	18 (64.3%)
1	10 (35.7%)
**Chemotherapy regimen**	
CHOP	23 (82.1%)
Others	5 (17.9%)
**Radiotherapy**	
Yes	8 (28.6%)
No	20 (71.4%)
**Prophylactic intrathecal injection**	
Yes	17 (60.7%)
No	11 (39.3%)
**LDH**	
≤ 220	17 (60.7%)
>220	11 (39.3%)
**β2-microglobulin**	
≤ 1.80	1 (3.6%)
>1.80	16 (57.1%)
Unknown	11 (39.3%)
**Site of lymphomatous involvement**	
Central nervous system	2 (12.5%)
Kidney	2 (12.5%)
Nasopharynx	3 (11.5%)
Lung	1 (6.3%)
Skin and subcutaneous tissue	4 (17.9%)
Adrenal gland	1 (6.3%)
Maxillary sinus	1 (6.3%)
Others	2 (12.5%)

*B symptoms, consist of unexplained fever above 38°C, night sweating, or weight loss of more than 10% within 6 months; IPI, International Prognostic Index; ECOG performance status, Eastern Cooperative Oncology Group performance status; CHOP, cyclophosphamide, doxorubicin, vincristine and prednisone; LDH, lactic dehydrogenase*.

During a median follow-up time of 39.2 months (range 20.5–107.7 months), 12 patients (30.9%) died. The median survival was 31.3 months. The cutoff values based on PFS for absolute neutrophil counts, absolute lymphocyte counts, absolute monocyte counts, absolute platelet counts, NLR, PLR, LMR, and SII were 3.03, 1.22, 0.54, 134.00, 2.49, 136.89, 3.39, and 428.40, respectively. The cutoff values based on OS were 3.03, 1.12, 0.55, 209.00, 2.66, 59.18, 3.31, and 428.40 for absolute neutrophil counts, absolute lymphocyte counts, absolute monocyte counts, absolute platelet counts, NLR, PLR, LMR, and SII, respectively. Patients were divided into two cohorts by these cutoff values.

### Univariate and Multivariate Analysis of PFS

**Figure 1:**
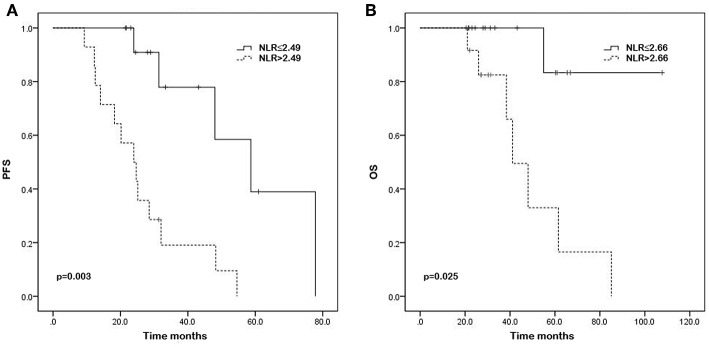
Kaplan–Meier curves of progression-free survival (PFS) **(A)** and overall survival (OS) **(B)** in testicular diffuse large B-cell lymphoma patients with high and low neutrophil/lymphocyte ratio (NLR) (*p* = 0.003, *p* = 0.025, respectively).

**Figure 2:**
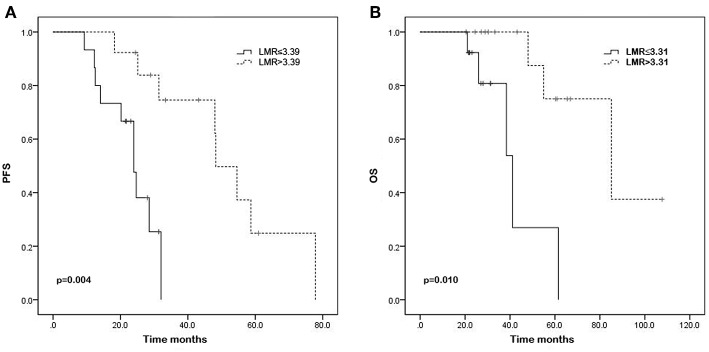
Kaplan–Meier curves of progression-free survival (PFS) **(A)** and overall survival (OS) **(B)** in testicular diffuse large B-cell lymphoma patients with high and low lymphocyte/monocyte ratio (LMR) (*p* = 0.004, *p* = 0.010, respectively).

The results of univariate analysis for PFS demonstrated that IPI score (28.7 vs. 24.3 months, *p* = 0.010), serum levels of LDH (31.4 vs. 21.7 months, *p* = 0.017), absolute neutrophil counts (28.0 vs. 25.1 months, *p* = 0.018), absolute monocyte counts (31.4 vs. 17.1 months, *p* = 0.001), NLR (30.2 vs. 24.3 months, *p* = 0.003), LMR (23.1 vs. 43.2 months, *p* = 0.004), and SII (30.2 vs. 24.3 months, *p* = 0.005) were significantly associated with PFS. The Kaplan—Meier survival curves show the same results (Figures 1, 2, and Supporting Figures 1–6). In the multivariate analysis for PFS, elevated absolute monocyte counts (hazard ratio [HR] 37.076; 95% confidence interval [CI] 4.691–293.037; *p* = 0.001) and NLR (HR 9.069; 95% CI 2.367–34.746; *p* = 0.001) were shown to be independently associated with an elevated risk for disease progression in patients with testicular diffuse large B-cell lymphoma. Table 2 shows the results of univariate and multivariate analyses for PFS.

**Table 2:** Univariate and multivariate analysis of PFS.

**Variable**	**Parameter**	**Median PFS (95% CI)**	**Univariate analysis**		**Multivariate analysis**	
			**HR (95% CI)**	***p* value**	**HR (95% CI)**	***p* value**
Overall	–	26.6 (25.0–37.8)	–	–	–	–
Age	≤ 60	25.1 (19.2–36.1)	1.00	0.742		
	>60	28.5 (24.4-43.4)	1.188 (0.426-3.310)			
Site of the primary tumor in the testicle	Left	23.5 (17.8–39.5)	1.00	–		
	Right	28.9 (25.7–43.1)	–			
	Bilateral	–	–			
Ann Arbor stage	I-II	28.0 (26.0–41.1)	1.00	0.698		
	III-IV	24.0 (17.2–40.8)	1.210 (0.462–3.169)			
B symptoms	Present	19.0 (−1.2–46.5)	1.00	0.126		
	Absent	28.3 (25.9–39.9)	0.363 (0.099–1.330)			
Extranodal involvement (excluding testicular)	0	30.2 (25.3–41.1)	1.00	0.174		
	1	24.3 (14.6–37.4)	0.936 (0.194–4.516)			
	>1	22.9 (−9.7–80.6)	2.451 (0.484–12.415)			
IPI score	0–1	28.7 (27.3–48.1)	1.00	**0.010**		
	2–5	24.3 (18.0–32.5)	4.052 (1.390–11.811)			
ECOG performance status	0	28.3 (26.3–45.0)	1.00	0.069		
	1	24.3 (18.7–29.0)	2.808 (0.924–8.531)			
Chemotherapy regimen	CHOP	28.0 (24.7–39.3)	1.00	0.633		
	Others	23.1 (9.0–49.0)	1.437 (0.325–6.364)			
Radiotherapy	Yes	35.9 (19.7–55.3)	1.00	0.752		
	No	24.9 (22.4–35.6)	1.185 (0.413–3.403)			
Prophylactic intrathecal injection	Yes	24.5 (21.6–40.9)	1.00	0.595		
	No	31.4 (23.0–40.6)	1.312 (0.481–3.575)			
LDH	≤ 220	31.4 (29.1–47.2)	1.00	**0.017**		
	>220	21.7 (16.4–25.8)	1.026 (1.280–12.664)			
Neutrophil counts	≤ 3.03	28.0 (23.1–47.7)	1.00	**0.018**		
	>3.03	25.1 (21.1–36.7)	6.044 (1.369–26.689)			
Lymphocyte counts	≤ 1.22	24.3 (18.4–33.2)	1.00	0.050		
	>1.22	28.5 (25.3–43.8)	0.357 (0.128–0.999)			
Monocyte counts	≤ 0.54	31.4 (26.7–44.6)	1.00	**0.001**	1.00	**0.001**
	>0.54	17.1 (12.4–21.9)	18.315 (3.351–100.093)		37.076 (4.691–293.037)	
Platelet counts	≤ 134.00	37.3 (6.9–81.8)	1.00	0.166		
	>134.00	24.6 (23.1–35.5)	4.185 (0.552–31.731)			
NLR	≤ 2.49	30.2 (27.3–47.7)	1.00	**0.003**	1.00	**0.001**
	>2.49	24.3 (17.7–33.0)	6.770 (1.913–23.960)		9.069 (2.367–34.746)	
PLR	≤ 136.89	25.1 (23.4–41.8)	1.00	0.061		
	>136.89	28.0 (19.6–39.6)	2.647 (0.956–7.327)			
LMR	≤ 3.39	23.1 (17.9–25.7)	1.00	**0.004**		
	>3.39	43.2 (32.0–53.1)	0.136 (0.035–0.538)			
SII	≤ 428.40	30.2 (26.0–46.3)	1.00	**0.005**		
	>428.40	24.3 (18.4–35.1)	6.091 (1.723–21.532)			

*IPI, International Prognostic Index; ECOG performance status, Eastern Cooperative Oncology Group performance status; CHOP, cyclophosphamide, doxorubicin, vincristine and prednisone; R-CHOP, CHOP plus rituximab; LDH, lactic dehydrogenase; NLR, neutrophil/lymphocyte ratio; PLR, platelet/lymphocyte ratio; LMR, lymphocyte/monocyte ratio; SII, systemic immune-inflammation index. The bold values indicate that p < 0.05 and the corresponding factors are significantly associated with survival*.

### Univariate and Multivariate Analysis of OS

High IPI score (45.6 vs. 30.8 months, *p* = 0.034), ECOG performance status of 1 (31.8 vs. 31.3 months, *p* = 0.016), high NLR (32.3 vs. 30.9 months, *p* = 0.025), and low LMR (26.6 vs. 51.5 months, *p* = 0.010) were associated with worse OS according to the results of univariate analysis. Significant associations were not observed between absolute neutrophil counts, absolute lymphocyte counts, absolute monocyte counts, absolute platelet counts, PLR, SII, and OS. The Kaplan—Meier survival curves were shown in the Figures 1, 2, and Supporting Figures 1–6. In the multivariable model, high LMR retained significance for favorable OS (HR 0.077; 95% CI 0.008–0.760; *p* = 0.028). ECOG performance status (HR 20.013; 95% CI 1.431–279.876; *p* = 0.026) was also proved to be independent predictor of OS (Table 3).

**Table 3:** Univariate and multivariate analysis of OS.

**Variable**	**Parameter**	**Median OS (95% CI)**	**Univariate analysis**		**Multivariate analysis**	
			**HR (95% CI)**	***p* value**	**HR (95% CI)**	***p* value**
Overall	–	31.3 (32.6–49.6)	–	–	–	–
Age	≤ 60	28.0 (23.5–40.9)	1.00	0.573		
	>60	41.1 (34.2–59.6)	1.861 (0.214–16.143)			
Site of the primary tumor in the testicle	Left	25.6 (19.7–50.5)	1.00	–		
	Right	41.1 (34.1–56.9)	–			
	Bilateral	–	–			
Ann Arbor stage	I–II	41.1 (32.6–54.2)	1.00	0.507		
	III–IV	30.3 (23.5–53.5)	1.607 (0.396–6.518)			
B symptoms	Present	28.7 (15.1–45.4)	1.00	–		
	Absent	32.4 (33.2–52.6)	–			
Extranodal involvement (excluding testicular)	0	30.2 (28.5–49.0)	1.00	0.780		
	1	36.2 (25.3–55.6)	1.794 (0.191–16.865)			
	>1	39.2 (−9.8–113.6)	2.366 (0.14–26.172)			
IPI score	0–1	45.6 (33.6–64.5)	1.00	**0.034**		
	2–5	30.8 (26.3–40.1)	10.597 (1.199–93.621)			
ECOG performance status	0	31.8 (32.3–57.6)	1.00	**0.016**	1.00	**0.026**
	1	31.3 (26.1–42.3)	14.838 (1.658–132.817)		20.013 (1.431–279.876)	
Chemotherapy regimen	R-CHOP	31.4 (33.2–52.6)	1.00	0.901		
	CHOP-like	23.1 (9.4–56.3)	1.146 (0.135–9.709)			
Radiotherapy	Yes	54.8 (32.2–81.9)	1.00	0.700		
	No	29.6 (28.1–41.4)	1.365 (020–6.649)			
Prophylactic intrathecal injection	Yes	28.9 (28.6–53.6)	1.00	0.171		
	No	33.3 (28.5–53.8)	2.400 (0.645–11.937)			
LDH	≤ 220	43.2 (36.9–61.1)	1.00	0.461		
	>220	26.0 (21.1–36.8)	1.909 (0.342–10.665)			
Neutrophil counts	≤ 3.03	31.2 (23.5–58.3)	1.00	0.200		
	>3.03	31.4 (31.1–51.4)	3.973 (0.482–32.735)			
Lymphocyte counts	≤ 1.12	44.6 (30.3–71.5)	1.00	0.110		
	>1.12	28.5 (28.6–48.3)	0.305 (0.071–1.308)			
Monocyte counts	≤ 0.55	41.1 (36.3–57.0)	1.00	0.549		
	>0.55	23.1 (21.2–28.0)	1239.773 (0.000–1.601E+13)			
Platelet counts	≤ 209.00	31.3 (30.2–50.0)	1.00	0.308		
	>209.00	44.5 (22.0–67.6)	0.027 (0.000–27.619)			
NLR	≤ 2.66	32.3 (30.3–56.2)	1.00	**0.025**		
	>2.66	30.9 (26.3–50.2)	11.186 (1.356–92.275)			
PLR	≤ 59.18	Constant	1.00	1.000		
	>59.18	31.4 (33.2–50.5)	1.00 (0.000–32153.454)			
LMR	≤ 3.31	26.6 (23.1–36.1)	1.00	**0.010**	1.00	**0.028**
	>3.31	51.5 (38.7–66.6)	0.106 (0.019–0.584)		0.077 (0.008–0.760)	
SII	≤ 428.40	31.3 (27.0–54.4)	1.00	0.106		
	>428.40	34.4 (29.6–53.5)	5.693 (0.692–46.812)			

*IPI, International Prognostic Index; ECOG performance status, Eastern Cooperative Oncology Group performance status; CHOP, cyclophosphamide, doxorubicin, vincristine and prednisone; R-CHOP, CHOP plus rituximab; LDH, lactic dehydrogenase; NLR, neutrophil/lymphocyte ratio; PLR, platelet/lymphocyte ratio; LMR, lymphocyte/monocyte ratio; SII, systemic immune-inflammation index. The bold values indicate that p <0.05 and the corresponding factors are significantly associated with survival*.

## Discussion

There is substantial evidence in various types of cancer that the host systemic immune response is a reliable independent prognostic factor, and pretreatment measurements of peripheral blood cells can be used to predict cancer outcomes. In this study, we reviewed 28 patients with testicular diffuse large B-cell lymphoma for their clinical and laboratory data, and demonstrated that IPI score, NLR and LMR were significantly associated with PFS and OS. LDH, absolute neutrophil counts, absolute monocyte counts, and SII were identified to be significantly associated with PFS; ECOG performance status was shown to be related to the risk of death. In addition, the results of multivariate analysis in this study revealed that absolute monocyte counts and NLR were independent prognostic markers for PFS, while ECOG performance status and LMR were proved to be independent predictors for OS in patients with testicular diffuse large B-cell lymphoma.

Lymphocytes have been reported to exert important roles in host's immune response and thus defense against development of cancer (16). In the past decades, neutrophils have been shown to promote tumor progression, and multiple mechanisms have been identified (17). Neutrophils secrete a variety of cytokines such as interleukin-2, interleukin-10, which is beneficial to cancer progression (18). The findings of this study indicated that elevated neutrophli counts are associated with high probability of cancer progression. Besides, increased neutrophil counts are related to high proportions of immature cells and thus influence functional status (17). Nevertheless, neutrophils were also have been reported to be potent anti-tumor effector cells (19). A number of recent studies have shown that high absolute neutrophil counts, low absolute lymphocyte counts, and high NLR in the blood as powerful immunologic prognostic parameters in patients with lymphomas (20, 21). Previous studies suggested that a prognostic parameter with HR > 2 is considered to be useful, which indicated that NLR was reliable to predict outcomes of testicular diffuse large B-cell lymphoma patients (22). However, further studies are needed to confirm the prognostic value of NLR among testicular lymphoma patients.

As regard to platelets, recent researches have reported that platelets promote tumor cell proliferation, dissemination, angiogenesis, and releasing of adhesion molecules and growth factors (23). Previous studies have also shown that platelets promote invasiveness of tumor cells through interactions between selectin, integrin and cancer cells (24). In addition, it has been shown that an increased absolute platelet counts might reflect degree of the systemic inflammation induced by tumor, because pro-inflammatory mediators such as interleukin-2, interleukin-3, and interleukin-6 can stimulate the proliferation of platelet progenitor cells (25). A limited number of studies have revealed that both high absolute platelet counts and PLR are strongly associated with poor survival in patients with lymphoma (26, 27). In the present study, neither absolute platelet counts nor PLR was found to be significantly associated with survival in patients with testicular diffuse large B-cell lymphoma. SII, involving platelet counts, neutrophil counts and lymphocyte counts, was found to be significantly associated with the risk of disease progression. Therefore, further researches are needed to investigate the ability of PLR and SII to predict survival in patients with testicular lymphoma.

The results of our study demonstrated that absolute monocyte counts and LMR were independent prognostic and predictive factors, which was previously reported in lymphoma and other types of cancer (28–30). Although cutoff values were slightly different in these studies, similar conclusions were drawn, indicating that an elevated LMR and low monocyte counts at diagnosis is associated with favorable outcomes. High monocyte counts have been reported to be related to increased number of bone marrow-derived myelomonocytic cells. Tsai et al. have reported that bone marrow-derived myelomonocytic cells can stabilize the tumor vasculature (31). Monocytes can also differentiate into tumor-associated macrophages, which release angiogenic factors and therefore promote angiogenesis (32). Therefore, absolute monocyte counts and LMR may serve as reliable prognostic factor for patients with testicular diffuse large B-cell lymphoma.

All the patients enrolled in this study received chemotherapy, and the most common regimen was CHOP. According to the results of univariate analysis, the survival of patients receiving CHOP was not significantly better than that of patients receiving other types of chemotherapy drugs. Previous studies have suggested that R-CHOP based chemotherapy with intrathecal chemotherapy may improve the rate of 5-year OS, which was not confirmed in our study, in part due to shorter follow-up duration (4). However, the results of our study demonstrated that patients receiving CHOP have better median PFS and OS. Furthermore, previous studies have suggested that radiotherapy and intrathecal chemotherapy may significantly improve OS (4). Our findings supported this conclusion, according to the HR value >1 for untreated patients. Therefore, in addition to surgery, we believed that chemotherapy, radiotherapy and intrathecal chemotherapy may also improve patient survival.

There are two potential limitations to this study. First, it was a retrospective, single-center study. Second, the population was somewhat small. Despite the above limitations, our study was the first to investigate the relationship between inflammation-based indices and prognosis in patients with testicular diffuse large B-cell lymphoma. Therefore, a multi-center, prospective study is required to confirm our results.

In conclusion, our study demonstrated that absolute monocyte counts, NLR and LMR were independent prognostic factors for PFS or OS in testicular diffuse large B-cell lymphoma patients. Since the indices incorporating blood cell counts are low cost parameters, they may provide additional prognostic value beyond standard clinicopathological parameters economically. However, further large-scaled studies are needed to confirm our results.

## Data Availability Statement

The datasets generated for this study are available on request to the corresponding author.

## Ethics Statement

The studies involving human participants were reviewed and approved by Ethics Administration Office of West China Hospital, Sichuan University. Written informed consent for participation was not required for this study in accordance with the national legislation and the institutional requirements.

## Author Contributions

JY designed the study, performed the data analysis, and drafted the manuscript. XG performed the data analysis and drafted the manuscript. JH, YD, and TZ participated in the data acquisition and drafted the manuscript. XM designed the study and revised the manuscript. All authors read and approved the final manuscript.

### Conflict of Interest

The authors declare that the research was conducted in the absence of any commercial or financial relationships that could be construed as a potential conflict of interest.
