# Supplementary material for: The Prognostic Value of Blood-Based Biomarkers in Patients With Testicular Diffuse Large B-Cell Lymphoma
Source: Front Oncol. 2019 Dec 10;9:1392. doi: 10.3389/fonc.2019.01392 (PMC6914857; doi:10.3389/fonc.2019.01392)
Supplement: Supplementary file 1 [file Image_1.pdf]

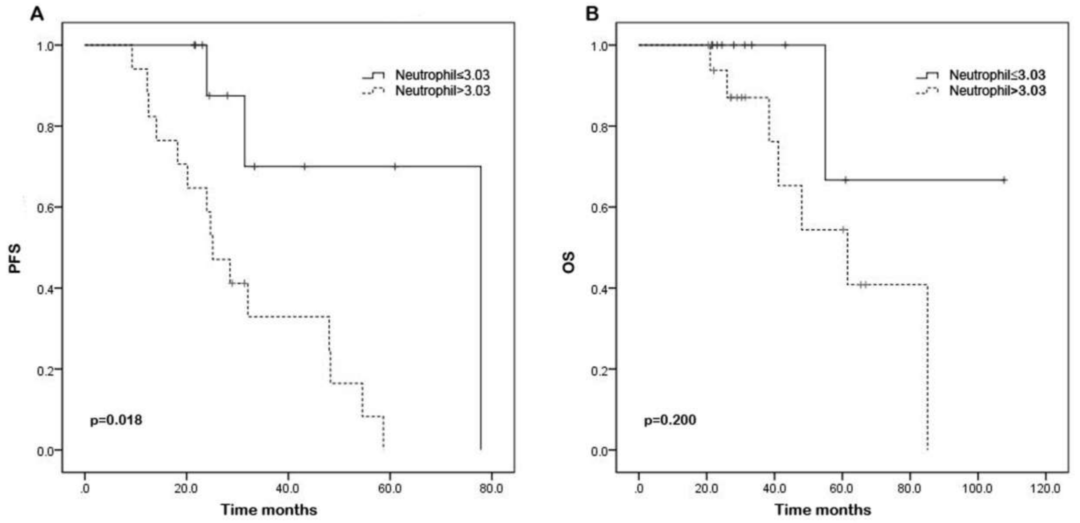

Supporting Figure 1: Kaplan–Meier curves of progression-free survival (PFS) and overall survival (OS) in testicular diffuse large B-cell lymphoma patients with high and low absolute neutrophil counts (p=0.018, p=0.200, respectively).

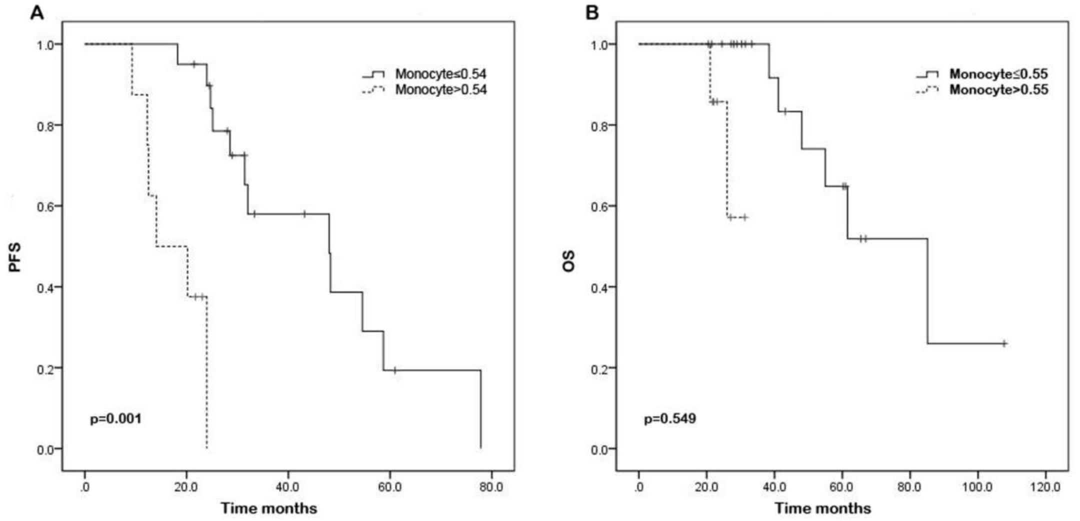

Supporting Figure 2: Kaplan–Meier curves of progression-free survival (PFS) and overall survival (OS) in testicular diffuse large B-cell lymphoma patients with high and low absolute monocyte counts (p=0.001, p=0.549, respectively).

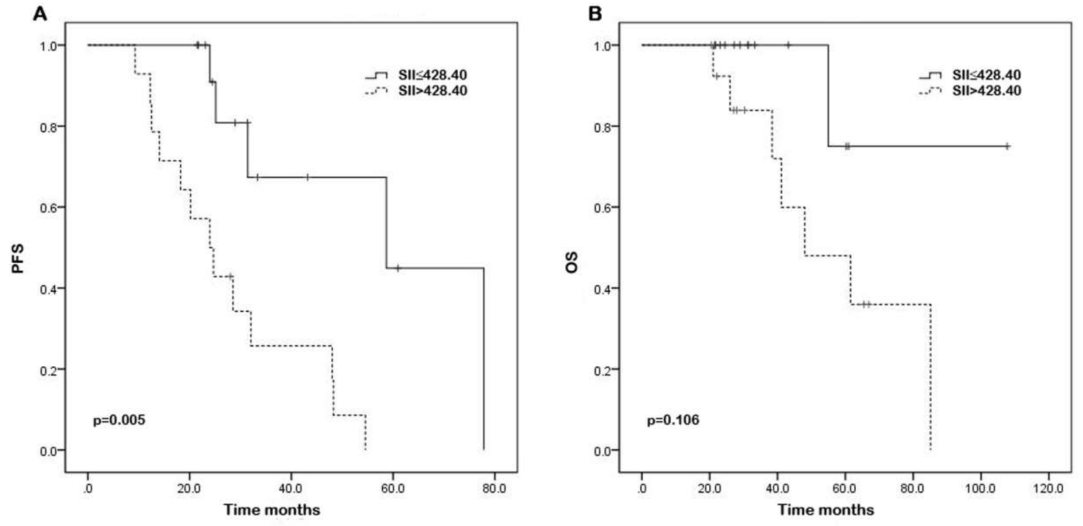

Supporting Figure 3: Kaplan–Meier curves of progression-free survival (PFS) and overall survival (OS) in testicular diffuse large B-cell lymphoma patients with high and low systemic immune-inflammation index (SII) (p=0.005, p=0.106, respectively).

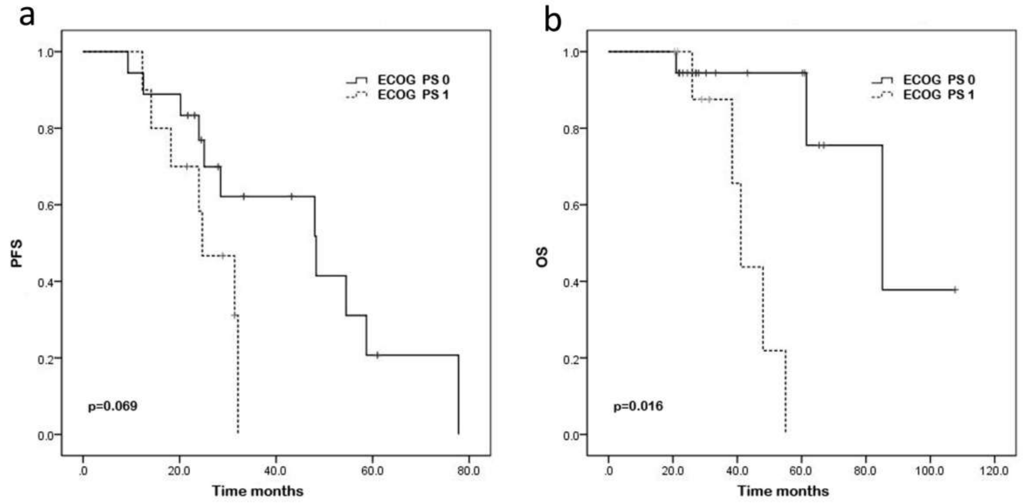

Supporting Figure 4: Kaplan–Meier curves of progression-free survival (PFS) and overall survival (OS) in testicular diffuse large B-cell lymphoma patients with Eastern Cooperative Oncology Group (ECOG) performance status (PS) of 0 or 1 (p=0.069, p=0.016, respectively).

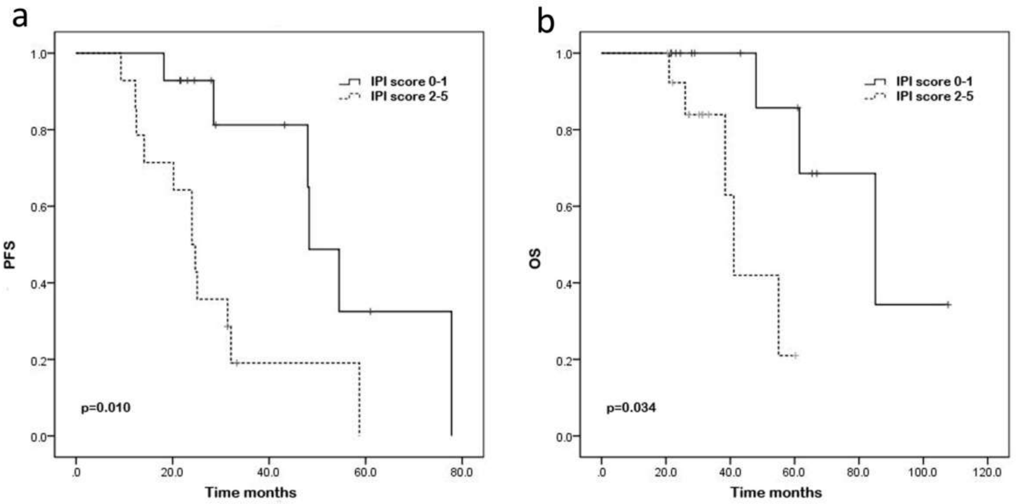

Supporting Figure 5: Kaplan–Meier curves of progression-free survival (PFS) and overall survival (OS) in testicular diffuse large B-cell lymphoma patients with International Prognostic Index (IPI) score 0-1 or 2-5 (p=0.010, p=0.034, respectively).

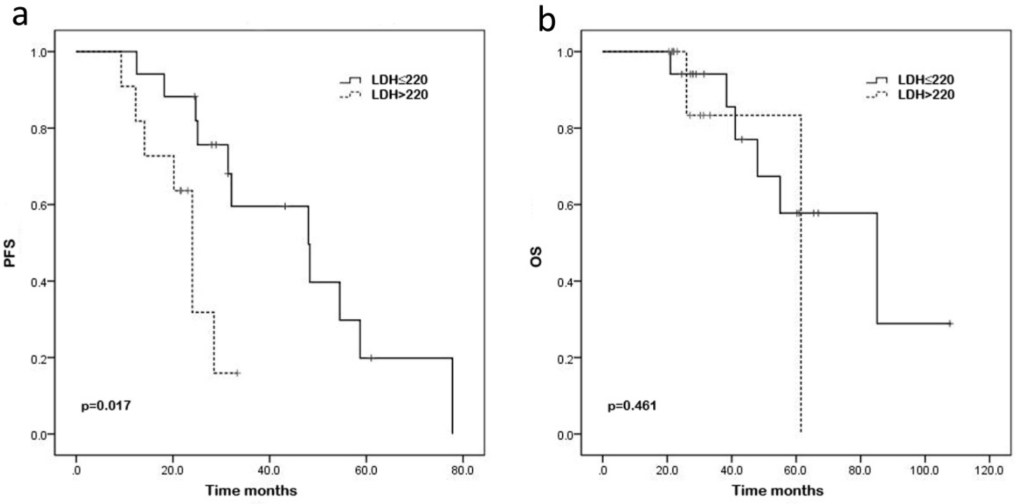

Supporting Figure 6: Kaplan–Meier curves of progression-free survival (PFS) and overall survival (OS) in testicular diffuse large B-cell lymphoma patients with high and low lactate dehydrogenase (LDH) (p=0.017, p=0.461, respectively).
